# The Progress of Hindooism in the United States

**Published:** 1889-02

**Authors:** Jos. Rodes Buchanan


### THE PROGRESS OF HINDOOISM IN THE UNITED STATES.

BY PROF. JOS. RODES BUCHANAN.

The word Theosophy has attained considerable currency of late, and the name Theosophist has been assumed by many as an expression of their faith. These are noble words—they imply divine wisdom, and if the votaries of divine wisdom are becoming numerous, it argues well for social progress. But, "all is not gold that glitters," and we shall find upon examination that much of what *is called Theosophy* has as little divine wisdom in it as any of the theological sects that stand in the way of human progress. To the word Theosophy I have no objections. I would prefer to be known myself, as a Theosophist,—a ruler of divine wisdom,—and I believe my name has been quoted by the president and founder of the Theosophic Society, as that of a Theosophist. Surely, no philosophic thinker can object to the broad and liberal definition of Theosophy given by Col. Olcott, "a system of free thought and universal fraternity, in the pursuit of the most elevated truths."

But, when we turn from these liberal professions and principles of the founder of the society to the actual practice, faith and teachings of his followers and to his own incidental admissions, we find that what is called Theosophy, instead of being a hard religion, philosophizes in just merely a resurrected Hindooism, as superstitious and theological a sect as any of those which bear the name of Catholicism, Calvinism, Shakerism, Mormonism, or Oshpheism, though less denunciatory and more tolerant. The whole theology and philosophy of ancient Hindoo writers is dumped wholly into the modern society, with its subdivision of the human soul into seven fragments, its mystical reincarnation and Karmer, and its denial or disregard of American spiritual science. There is no more of Theosophy in this than in any other superstitious and sectarian movement; and though I might recognize in some of the leading minds of the society a true Theosophic spirit, I cannot recognize in the general drift of what is presented as Theosophy, anything more than a revival of that superstitious spirit which would bind the growing present to the stationary past—which is the essence of all religious superstitions.

American Theosophy is not disposed to bind itself to the dead body of the past of either Palestine, India, or Arabia. True Theosophy cannot recognize in any presentation of Hindooism anything more than a resurrection of the follies and mysticisms of the juvenile period of the race. Catholicism, too, has its schools of philosophy, as well as Hindooism, and has as much pretention to sublime wisdom. But it is a maxim with all truly liberal and progressive thinkers that correct knowledge upon any subject is to be found only in the results of the accumulated experience and untrammelled investigation of modern times, while ignorance and delusion are found in greater and greater abundance the further back we go toward the ages of barbarism, even though that may be pretentiously called the wisdom-age.

Hindooism, like all other sectarianisms, requires us to believe that the highest wisdom has been hidden in the dark past, that antidates authentic history, but really to seek for any scientific philosophy in that direction is almost as hopeless an undertaking as to hunt in the pyramids of Egypt for the electrical science developed by such as Faraday and Edison. We may find ethical speculation and a few instructive perceptions, but very little worthy of the name of science, unless it be astronomical or astrological.

It was a very skillful plan to introduce Hindooism under the attractive name of Theosophy. But is it Theosophy which constitutes the staple commodity of what are called Theosophic societies and Theosophic writers? I think if the reader will inquire he will find about as little Theosophy as in any liberal Christian church, and far less than in any spiritual writing. Reincarnation, Karma, elementaries, shells, Astral mysteries and mysterious Oriental words, and still more mysterious, purposeless and pointless speculations, are the chief stock in trade of what *is called* Theosophy; and I would respectfully submit the question whether it would not be more just to science and to the public, who are easily deceived, to lay aside the word Theosophy and use the more appropriate title of Hindooism, since it is fast assuming the character of Buddhism. Let us, therefore, in speaking of these things ourselves, pay some respect to the proprieties of the English language and use the word Hindooism for that which has *assumed* the name of Theosophy.

Col. Olcott is welcome to eulogize Lord Buddha as the highest exemplar of wisdom and virtue, but I beg leave as an American Theosophist, to differ considerably from his roseate view of the subject, and I object decidedly to a professedly creedless society becoming the active propagandist of Hindooism; for I cannot recognize in Sakya Muni either a true philosopher or a very wise ethical teacher, and I regret that Col. Olcott's fine talents have been devoted to ruining the spirit of antiquity. No glamour of ancient renown or multitudinous following of Asiatic fanatics can make the morbid and unnatural character of Buddha a suitable model for enlightened men of the nineteenth century. It may suit the ascetic monk. It may even come into sympathy with the pessimitic Schopenhauer, but it has little of the disinterested energy and heroic love which form the character of Christ, and which are the inspiration of a truly noble and beneficent life. I don't think we gain anything ethically by substituting "Lord Buddha" for "Lord Jesus," or by substituting *ancient authority* for *modern science.*

The principles avowed by Col. Olcott, the head of the society, are unsectarian. He says: "We first proclaim the universal brotherhood of man and the duty of all to join in what will promote the welfare of the human race, especially those who are weakest and most need help. There have been true Theosophists in every creed, true seers who have lifted the secret veils of nature and penetrated her mysteries."

But as every movement tends to degenerate from the ideal of its founder, the Theosophic societies seem to have degenerated into a mere propaganda of Hindooism, and the study or cultivation of Theosophy is understood to mean a cultivation of that which constitutes Hindooism—its acceptance as a matter of faith. It is not understood to mean the cultivation of psychic science, but rather an abandonment of experimental investigation in modern methods, and surrender to unquestioning faith in the antique notions of India. Hence, I am disposed to speak of the movement in the United States as a propaganda of Hindooism.

The more we reflect upon it the more clearly it appears that Hindooism, though introduced under the graceful cloak of Theosophy, is not Theosophic, but is as widely distinct from and opposed to genuine psychic, or Theosophic science and *enlightened* benevolence, as the Roman Catholic Church, which, like the Oriental, recognizes the psychic phenomena, but ascribes them to misleading spirits as the Hindoo ascribes them to shells and black magic. All forms of sectarianism alike discourage and discredit the free psychal science, the genuine Theosophy in which America has taken the lead. The abuses of spiritualism by fraud, by abnormal mediumship and credulous follies have nothing to do with the noble forms of science which are here developed, which bring psychic science into connection with the great body of positive sciences concerning man, which are included in the comprehensive word Anthropology.

